# A *Saccharomyces eubayanus* haploid resource for research studies

**DOI:** 10.1038/s41598-022-10048-8

**Published:** 2022-04-08

**Authors:** Jennifer Molinet, Kamila Urbina, Claudia Villegas, Valentina Abarca, Christian I. Oporto, Pablo Villarreal, Carlos A. Villarroel, Francisco Salinas, Roberto F. Nespolo, Francisco A. Cubillos

**Affiliations:** 1 ANID-Millennium Science Initiative-Millennium Institute for Integrative Biology (iBio), 7500574 Santiago, Chile; 2 Departamento de Biología, Facultad de Química y Biología, Universidad de Santiago de Chile, 9170022 Santiago, Chile; 3 ANID-Millenium Nucleus of Patagonian Limit of Life (LiLi), 5090000 Valdivia, Chile; 4 Instituto de Ciencias Biológicas, Universidad de Talca, 3465548 Talca, Chile; 5 Instituto de Investigación Interdisciplinaria (I3), Universidad de Talca, 3465548 Talca, Chile; 6 Facultad de Ciencias, Instituto de Bioquímica Y Microbiología, Universidad Austral de Chile, 5090000 Valdivia, Chile; 7 Facultad de Ciencias, Instituto de Ciencias Ambientales y Evolutivas, Universidad Austral de Chile, 5090000 Valdivia, Chile; 8 Center of Applied Ecology and Sustainability (CAPES), 8331150 Santiago, Chile

**Keywords:** Biotechnology, Genetics, Microbiology, Molecular biology

## Abstract

Since its identification, *Saccharomyces eubayanus* has been recognized as the missing parent of the lager hybrid, *S. pastorianus*. This wild yeast has never been isolated from fermentation environments, thus representing an interesting candidate for evolutionary, ecological and genetic studies. However, it is imperative to develop additional molecular genetics tools to ease manipulation and thus facilitate future studies. With this in mind, we generated a collection of stable haploid strains representative of three main lineages described in *S. eubayanus* (PB-1, PB-2 and PB-3), by deleting the *HO* gene using CRISPR-Cas9 and tetrad micromanipulation. Phenotypic characterization under different conditions demonstrated that the haploid derivates were extremely similar to their parental strains. Genomic analysis in three strains highlighted a likely low frequency of off-targets, and sequencing of a single tetrad evidenced no structural variants in any of the haploid spores. Finally, we demonstrate the utilization of the haploid set by challenging the strains under mass-mating conditions. In this way, we found that *S. eubayanus* under liquid conditions has a preference to remain in a haploid state, unlike *S. cerevisiae* that mates rapidly. This haploid resource is a novel set of strains for future yeast molecular genetics studies.

## Introduction

Model organisms have been used for decades in biological and molecular studies, and are central players in the major breakthroughs in biology^1^. In eukaryotes, the yeast *Saccharomyces cerevisiae* is a popular model organism for research across many fields, ranging from classical genetics, molecular biology, biochemistry, and cellular physiology to population and comparative genomics^2^. The widespread utilization of *S. cerevisiae* is primarily because of its short cell cycle, small genome size, high recombination rates and self-fertilization capacity^3^. These features allowed researchers to generate different tools to simplify the genetic manipulation of yeast, such as in the large-scale development of null mutants^4–7^, and the generation of haploid and diploid strains with distinctive auxotrophic markers in different genetic backgrounds^8–11^. The generation of these collections was prompted by the easy manipulation of conventional genetic recombination approaches, and took advantage of the high homologous recombination (HR) rate in *S. cerevisiae*^12,13^. However, these approaches require the utilization of selective markers, drug-selectable markers and/or auxotrophic nutritional markers^14,15^. The use of auxotrophic markers can cause fitness changes, confounding the phenotypic effects of gene deletion^15,16^. Furthermore, there is a limited number of drug-selectable markers, restricting the simultaneous study of the fitness effects in null-mutants of multiple genes^15^. Although these selective markers can be recycled by recombinase-based methods such as Cre/LoxP or Flp/FRT, these techniques are time-consuming, since additional transformation rounds are required to remove selective markers^14,17,18^. In addition, these techniques have the drawback of leaving a copy of a repeat sequence in the genome, which can generate genome instability after multiple rounds of marker recovery^14,19^. An alternative to these laborious procedures is the type II bacterial Clustered Regularly Interspaced Short Palindromic Repeats (CRISPR) associated to the Cas9 protein (CRISPR-Cas9 system), allowing targeted double-strand breaks (DSBs) and simultaneous gene editing of all copies of the target sequence^14,20,21^. Since HR is the dominant mode of DSB repair in *Saccharomyces* yeast, short single-stranded- or double-stranded-DNA donor oligos can be used as a repair template, allowing targeted genome edition^14^. Different studies have precisely designed point mutations, single and multiple gene deletions, and multiplexed genome modifications at different *loci,* representing an ideal system for targeted mutagenesis^20,22,23^. In this sense, the CRISPR-Cas9 system has the advantage of both ease of use and broad applicability.

The *S. cerevisiae* collections have prompted different genetic experiments, such as the description of a gene’s function^7^, quantitative trait loci (QTLs) analysis and validation^24–27^, and gene expression analysis^28,29^. However, *S. cerevisiae* only represents a small fraction of the overall genetic and phenotypic diversity in the *Saccharomyces* genus^30^. Therefore, recent bioprospecting efforts have described large sets of strains in other *Saccharomyces* species, shifting the focus towards novel population genomics studies, including genome evolution, ecology and biotechnology. Moreover, novel *Saccharomyces* strains have a potential utilization in fermentation processes, using intra- or interspecies hybrids, and so these organisms are of interest for large-scale phenomics studies^30–33^. For example, during the past decade, *Saccharomyces eubayanus* and *Saccharomyces jurei* have been described as novel species in the *Saccharomyces* genus, where both species exhibit a great potential for brewing and dough fermentation^34–40^.

*S. eubayanus* has gained relevance since its isolation and identification in 2011 in the Argentinian Patagonia, as it is the missing parental species of the allopolyploid hybrid *Saccharomyces pastorianus*^41^. This hybrid is responsible for lager beer fermentation, and is currently used for the production of more than 90% of global commercial beer^42^. However, lager strains have a limited genetic diversity, which translates into a reduced variety of lager beers and low organoleptic differentiation^31,43^. Therefore, the isolation of *S. eubayanus* represented a promising step towards the generation of new brewing strains, either directly or through the generation of new *Saccharomyces* hybrids. Recently, several groups have reported the isolation of different *S. eubayanus* strains, including in China^44^, North America^45^, New Zealand^46^, and more recently, in Chile^36^. Phylogenetic analysis showed that this species has a wide genetic diversity, with five different lineages, where those from Patagonia have the most marked global genetic diversity compared to Holarctic and Chinese lineages^36,37,47,48^. Furthermore, this genetic diversity translates into an interesting phenotypic diversity, including phenotypes related to brewing conditions^34,36^. In this context, considering that *S. eubayanus* has been isolated from wild environments, and to date, there is no evidence of *S. eubayanus* isolation from fermentative environments, this yeast is thus an interesting candidate to become a valuable organism for evolutionary, ecological and genetic studies^32^. However, in order to adequately use *S. eubayanus* in molecular genetic studies, it is necessary to generate suitable genetic tools to facilitate its manipulation in the laboratory. Strains of *S. eubayanus* are diploid, highly homozygous and probably homothallic^36,49^. Thus, the generation of stable haploid versions representative of a broader genetic diversity in the species is an important step towards promoting the study of this biotechnologically relevant microorganism. In this sense, previous studies generated heterothallic Holarctic and admixed North American *S. eubayanus* strains for different biological studies^50–52^.

In this study, we developed a collection of 57 isogenic haploid strains representative of three Patagonian lineages described in the species. For this, we deleted the *HO* locus using the CRISPR-Cas9 system. The selected haploid strains from both mating types generated in this study were characterized under different conditions, observing that most spores mimic the diploid parental phenotype. Moreover, we sequenced a whole tetrad using nanopore sequencing to identify possible Cas9 off-targets and meiotic rearrangements. Finally, as proof of concept, we performed two experimental approaches for the generation of intraspecific hybrids under rich media, finding striking differences in mating frequency compared to *S. cerevisiae*. This new collection of *S. eubayanus* haploid strains is a valuable community resource that complements previously generated for molecular genetics studies.

## Results and discussion

### Generation of a large collection of haploid derivates in *S. eubayanus*

**Figure 1:**
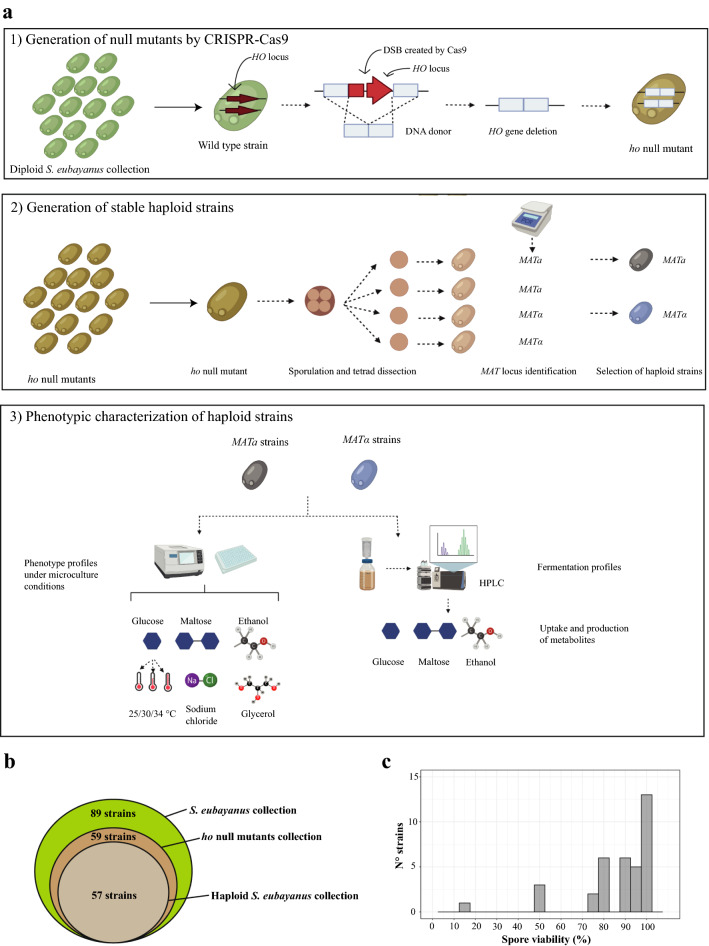
Generation of a large set of stable haploid *S. eubayanus* strains. (**a**) Experimental procedure designed to generate the set of stable haploid *S. eubayanus* strains. A1. *HO* deletion, A2. Dissection of stable haploids and A3. Phenotyping of haploid strains. (**b**) Number of strains present in the wild collection of *S. eubayanus*, the sub-sets of null mutants and haploid strains generated (Created with BioRender.com). A single ‘*MATa’* and ‘*MATα’* spore was selected for phenotyping (**c**) Histogram of spore viability in 36 successfully transformed isolates (*Δho*).

*S. eubayanus* is a diploid yeast, homothallic, and has been isolated mainly from wild environments^36,49^. To generate heterothallic strains, we decided to knock out the *HO* gene in wild diploid strains from different lineages (Fig. 1a). The *HO* gene encodes a site-specific endonuclease that promotes mating-type switching from a to α, or vice versa, in haploid strains, and its deletion has been widely used for the generation of stable haploid cells in yeast^8,11,53^. To delete the *HO* gene, we used a plasmid expressing the Cas9 enzyme and a gRNA cloning site developed by Fleiss et al*.*^54^; however, this plasmid had only been used in *S. cerevisiae*, without evaluating its efficiency in other *Saccharomyces* species.

We first evaluated the efficiency of the CRISPR-Cas9 system and the plasmid pAEF5 to obtain null mutants for the *HO* gene in the CL216.1 strain of *S. eubayanus* using four different gRNAs (gRNA1, gRNA2, gRNA3 and gRNA4, see methods), together with a 100-bp double stranded repair DNA fragment. *HO* gene deletion was observed in 11%, 0%, 42% and 0% of the investigated colonies for each gRNA (from 1 to 4), respectively. The deletion or mutation efficiencies obtained for *S. eubayanus* using CRISPR-Cas9 varies between studies, depending on the targeted region. In some cases, 100% efficiency was achieved in generating the targeted mutations (at the *MALTX* genes^55^), whilst in others, the efficiencies varied between 3 and 40%, depending on the gene and the genetic background^56^. Also, diploid yeast cells exhibit a barrier in utilizing DSB-mediated transformation schemes because diploid cells prefer homologous chromosomes for DSB repair, rather than exogenous linear DNA, resulting in a minority of recovered strains acquiring the targeted modification^10^. However, the efficiencies obtained in our study are in agreement with the efficiencies reported elsewhere in different *S. cerevisiae* strains utilizing the CRISPR-based gene deletion approach (1.3–100%)^21,23,57^. Considering the diploid nature of *S. eubayanus*, CRISPR-Cas9 based strategies allowed us to mutate both alleles in a single transformation event without the need to insert a selection cassette into the genome^20^. CRISPR-Cas9 has been used successfully in a few studies in *S. eubayanus*, generating null mutants^55^, integrating and overexpressing genes^58^, and introducing loss-of-function SNP mutations^56^. Therefore, based on our initial findings, gRNA3 was selected to repeat this procedure in a greater number of strains.

We subsequently used a collection of *S. eubayanus* strains spanning three of the six main lineages in the species, together with a subset of admixed strains, comprising about 55% of the genetic diversity found in this yeast species^36,37^. In this way, the initial collection of strains for *HO* knockout considered 89 strains (Fig. 1b), of which 83 strains were previously isolated from 10 different locations in Chile belonging to the PB-1, PB-2 and PB-3 populations; admixed clusters (ADM), five North American strains that grouped in the PB cluster, and the reference strain from Argentina were also used (Supplementary Table S1). Overall, 59 of the 89 *S. eubayanus* isolates were successfully transformed with the plasmid pAEF5 (Fig. 1b), including 20 strains from PB-1, 11 from PB-2, 19 from PB-3 and nine ADM strains (Supplementary Table S1). *S. eubayanus* has been genetically modified using electroporation and the lithium acetate procedure, however, historically only three different genetic backgrounds have been mainly used^55,56,58^. In this context, this study achieved genetic editing on a broader panel of genetically-distinct *S. eubayanus* strains.

To obtain stable haploid derivates, transformants were sporulated and dissected by micromanipulation (Fig. 1a). Only tetrads with four viable spores were considered for *MAT* locus genotyping. In this way, we obtained *MATa* and *MATα* haploid versions for 57 out of the 59 parental strains containing the plasmid pAEF5 (Fig. 1b, Supplementary Table S1). Strains CL1002.1 and CL611.1 did not sporulate and we were unable to obtain haploid derivates. Furthermore, in strains CL704.2 and CL836.1 we did not obtain four viable spores from the same ascus; however, as we identified *MATa* and *MATα* versions in both cases, both were included in the final haploid collection. Finally, we evaluated spore viability in 36 out of the 57 successfully transformed isolates, obtaining spore viabilities between 15 and 100% (Fig. 1c). In all, only one isolate exhibited spore viabilities lower than 50%, six isolates presented spore viabilities between 50 and 75%, and 30 isolates had spore viabilities over 75%. The high proportion of strains able to sporulate (~ 97%) agrees with previous data observed in *S. eubayanus*^41^ and other wild *Saccharomyces* strains, for which high sporulation and spore viability levels have been reported^8,59,60^.

### Phenotypic profiling demonstrates similar phenotypes among diploid and haploid strains

**Figure 2:**
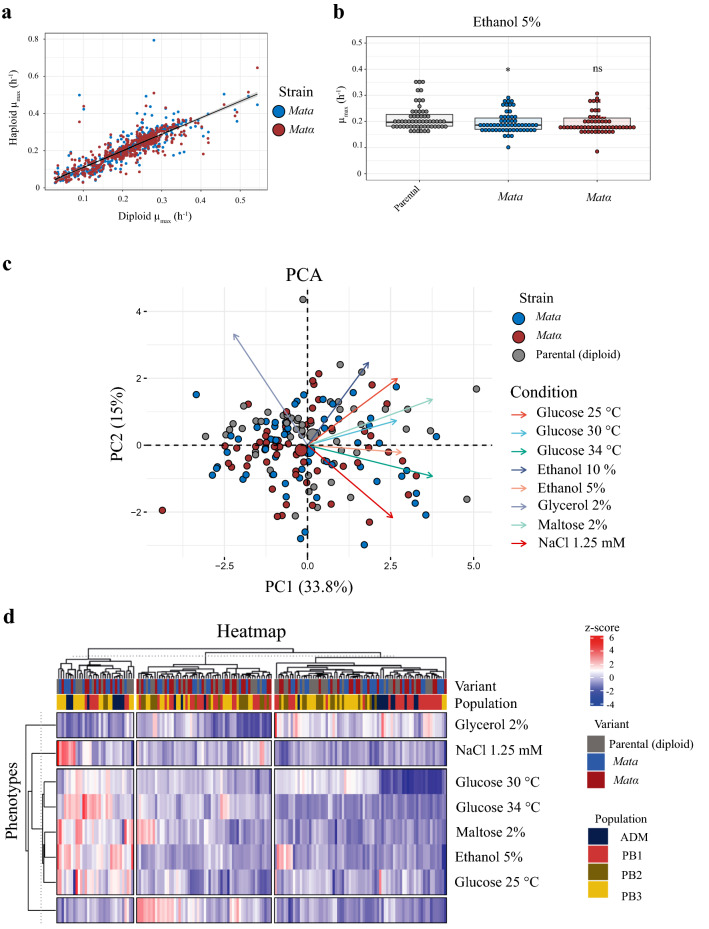
Phenotypic characterization of haploid strains and their respective diploid parental strains. (**a**) Maximum specific growth rates of diploids and haploids comparing all strains, ploidy, and environments. Line indicates the 1:1 correlation. (**b**) Maximum specific growth rates for diploids and haploids for all strains under 5% ethanol. Plotted values correspond to mean value of each strain. (*) depicts different levels of significance between haploid and diploid strains (Student t-test; *p ≤ 0.05 and ns: non-significant). (**c**) Principal component analysis (PCA) using the maximum specific growth rates under eight growth conditions, together with the distribution of individual haploid and diploid strains. Arrows depict the different environmental conditions (**d**) Hierarchically clustered heatmap of phenotypic diversity within haploid and diploid parental strains. Phenotypic values are calculated as normalized z-scores.

For each strain we characterized the phenotypic profile of one *MATa* and *MATα* haploid derivates and their parental strains under eight different microculture conditions, including: 2% glucose at 25, 30 and 34 °C; 1.25 mM NaCl; 2% maltose; 5% and 10% ethanol; and 2% glycerol (Supplementary Table S2). In general, we observed that for most conditions and genetic backgrounds, there were no statistically significant differences in maximum growth rates between haploid strains and their respective parental strain (Fig. 2a). In this sense, we observed that in *S. eubayanus* most haploids and diploids exhibited similar performance under the environmental conditions tested in this study, where no general fitness advantages were evident in either ploidy state. Previously reported observations in *S. cerevisiae* and *S. paradoxus* using a more extensive set of conditions and strains also demonstrated no differences between either haploidy or diploidy^61^. However, we did find certain cases where one or both haploid versions showed statistically significant differences under certain conditions (Supplementary Table S2). We found specific ploidy x environment interactions, particularly for ethanol tolerance, where *Mata* haploid strains exhibited significantly lower growth rates than diploid strains (*p*-value < 0.05, Fig. 2b). The trend for the opposite mating type version *MATα* was similar, however, we found marginally significant differences in this case (*p*-value = 0.06, Student t-test). Indeed, DNA damage agents were previously shown to favor diploid asexual proliferation in *S. cerevisiae* and *S. paradoxus* compared to haploid strains^61^.

To analyze the whole phenotypic landscape in the set of 57 diploid and haploids strains, we performed a PCA analysis (Fig. 2c). The PC1 and PC2 components explain 33.8% and 15% of the observed variance, respectively, and combined, the two components account for 48.8% of the overall variation. PCA shows that growth in glycerol correlates negatively with the rest of the phenotypes where the carbon source was glucose or maltose. Interestingly, the individual factor map indicates no significant separation pattern according to ploidy and/or haploid strain mating-type, suggesting comparable phenotypes among diploid and haploid strains.

In addition, we also performed hierarchical clustering of the phenotypic data (Fig. 2d), observing three main clusters. Notably, 60% of the parental strains (34) clustered together with their two haploid versions, 18% (10) of the diploid parents clustered with one of the haploid versions, while 23% (13) distributed across different clusters compared to their two haploid versions. These results corroborate that most of our haploid strains are representative of their parental genetic backgrounds, as previously described^61^. For example, the CL715.1 strain and its haploid variants did not show statistically significant differences in seven of the eight media evaluated. However, the alpha version showed statistically significant differences when cultivated in 10% ethanol (Supplementary Table S2). Phenotypic differences between haploid and parental strains have also been observed in other yeast collections^9,11^. Therefore, this extensive collection of *S. eubayanus* represents a valuable resource for molecular genetics studies.

### Similar brewing fermentative profiles in haploid and diploid strains

**Figure 3:**
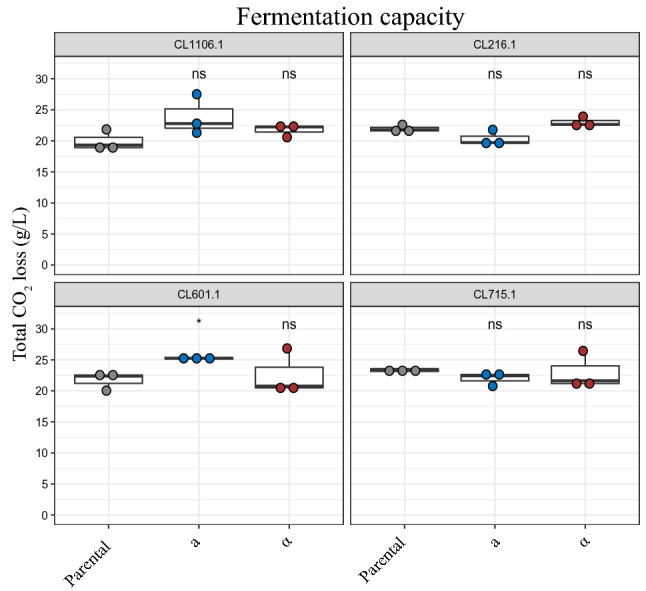
Fermentation performance of haploid and diploid strains. Total CO_2_ loss for CL1106.1, CL216.1, CL601.1 and CL715.1 strains and their haploid strains. Plotted values correspond to three biological replicates. The (*) represents different levels of significance between the phenotype of haploid strains and their respective parental strain (t-test; *p ≤ 0.05 and ns: non-significant).

Considering the relevance that *S. eubayanus* has gained in the last decade for lager beer fermentation, we evaluated the fermentative profiles of parental strains and their haploid versions under conditions of beer wort fermentation. For this, we selected four strains representative of different lineages (CL1106.1 (ADM), CL216.1 (PB-3), CL601.1 (PB-3) and CL715.1 (PB-2)) for a 15-day lager fermentation in a 12°Brix wort and monitored the fermentation outcome by estimating CO_2_ loss. Overall, we did not observe statistically significant differences in the total CO_2_ output between haploid strains and their respective parental strains, except for the CL601.1-a strain (Fig. 3). All strains completely consumed the sugars present in the beer wort, except for maltotriose, which was previously reported as not being metabolized by *S. eubayanus*^62^. The haploid strains of CL216.1 and CL715.1-a showed significantly higher ethanol production than their respective parental strains, with differences of 0.74% (t-test, *p* < 0.001) and 0.64% (t-test, *p* < 0.001) for CL216.1-a and CL216.1-α strains, respectively, and 0.48% (t-test, *p* < 0.05) for the CL715.1-a strain (Supplementary Table S3). In general, the results of the fermentation assay demonstrate that this set of haploid strains is representative of the phenotypic fermentative performance of their diploid parents. Obtaining phenotypically representative haploid variants of the wide diversity of *S. eubayanus* strains is of relevance for genetic and biotechnological studies. In particular, these strains can be used at the fermentative level, where genetic analyses explaining differences between strains at the phenotypic level have not been undertaken. For example, strains belonging to the PB-3 subpopulation have a higher fermentative capacity than strains belonging to PB-2 or PB-1^36^, reaffirming the existence of natural genetic variants in wild strains resulting in phenotypic diversity. In this way, this collection of haploid strains represents a valuable tool to be considered, for example, in the identification of QTLs.

### Low levels of off-target mutations in sequenced haploid strains

Previous studies have described off-target effects resulting from the use of a CRISPR-Cas9 system in genome editing^63^. In this sense, to evaluate off-target mutations, we sequenced the genome of three haploid strains, i.e., one spore of CL715.1, CL216.1, and CL601.1 (strains previously evaluated under fermentation conditions), using high-coverage whole-genome sequencing (WGS, Supplementary Table S4). After mapping the reads to the *S. eubayanus* reference genome (CBS12357)^55^, we performed variant calling and compared each haploid strain with their corresponding wild-type genotype that had been previously obtained using WGS^36^ to discover novel off-target mutations. We initially found 6, 12, and 16 putative mutations in CL601.1, CL216.1, and CL715.1, respectively (Supplementary Table S5a). After manual inspection of the alignments, we confirmed two single-nucleotide mutations, both occurring in the CL216.1 haploid strain in chromosomes 4 and 8. Interestingly, the mutation located in chromosome 4 was upstream of the *HO* gene, within the donor DNA recombination region, where the new allele coincides with the reference sequence used to generate the donor DNA. Furthermore, the mutation at chromosome 8, located in the coding sequence of the *PRO2* gene, was also observed in the CL216.1 diploid sequencing using long reads^35^, suggesting that this mutation arose before the CRISPR-Cas9 procedure, and was not detected in the original CL216.1 WGS^36^. Off-target mutations correspond to DNA modifications at unintended sites, such as SNPs, deletions, insertions or inversions^63^, and depend on gRNA and experimental conditions^64^. In *Saccharomyces* yeast, these off-target mutations could arise from NHEJ of endonuclease-mediated DSBs, by HR between the 100 bp DNA donor or by homologous sequences at other genomic locations similar to the target sites^57^. Studies have evaluated the presence of off-target mutations after genome editing in *Saccharomyces* yeasts, in which they have been shown to be rare in haploid or homozygous strains^56,57,65,66^. Furthermore, off-target effects are considered unlikely in a small genome such as that of yeast^21^, coinciding with the results obtained in our study.

We also used haploid WGS to validate heterozygotes sites previously determined after WGS of diploid strains^36^. Strikingly, we found that out of the 807, 720 and 942 heterozygous sites called after WGS of the diploid strains CL216.1, CL601.1 and CL715.1, respectively, 98% of them were again identified as heterozygous sites in the haploid strains (Supplementary Tables S5b, S5b and S5b), suggesting that most heterozygote calls in diploids were false positives, and do not represent real heterozygotes sites, but rather polymorphic repeated regions or technical sequencing artifacts. These results suggest that the CRISPR-Cas9 technique used in our study likely generated a low number of off-targets in the manipulated strains. These results should be confirmed by sequencing a more significant number of haploid derivates..

Although we found few heterozygous sites validated after WGS of the three haploid strains (16–29) (Supplementary Tables S5b, S5b and S5b), meiotic events could also lead to the generation of structural variants and/or chromosome mis-segregation. Hence, we sequenced each of the four spores corresponding to one complete tetrad of the CL216.1 strain using nanopore long-read sequencing (Supplementary Table S6). Long-reads further confirmed the low number of heterozygous sites occurring in *S. eubayanus*, limited to only one site that segregated in a clear 2:2 proportion among spores (Supplementary Fig. S1). The draft genomes of each spore showed the absence of structural variants among spore genomes and demonstrated a flawless meiotic event (Supplementary Fig. S2). In general, meiosis provides a large source of genetic diversity by rearranging allelic combinations^67^, which is important to consider when haploid strain collections are generated. The main source of genetic diversity is meiotic crossovers (COs), that are reciprocal exchanges of chromosome arms between homologous chromosomes and are crucial for accurate homolog segregation at meiotic division I, and their absence leads to mis-segregation of homologs and aneuploid gametes^68^. Another source of genetic diversity during meiosis is the generation of punctual mutations. These normally occur in meiosis at higher frequencies than in vegetatively grown cells of *S. cerevisiae*, as a result of DSB repair in meiosis during the recombination process^69^. In this sense, we did not observe aneuploid or structural variants in any of the four spores derived from the CL216.1 strain, or de novo mutations, suggesting correct COs and homolog segregation during the meiotic process in CL216.1.

### Intra-specific hybridization is common in *S. cerevisiae*, but not in *S. eubayanus*

**Figure 4:**
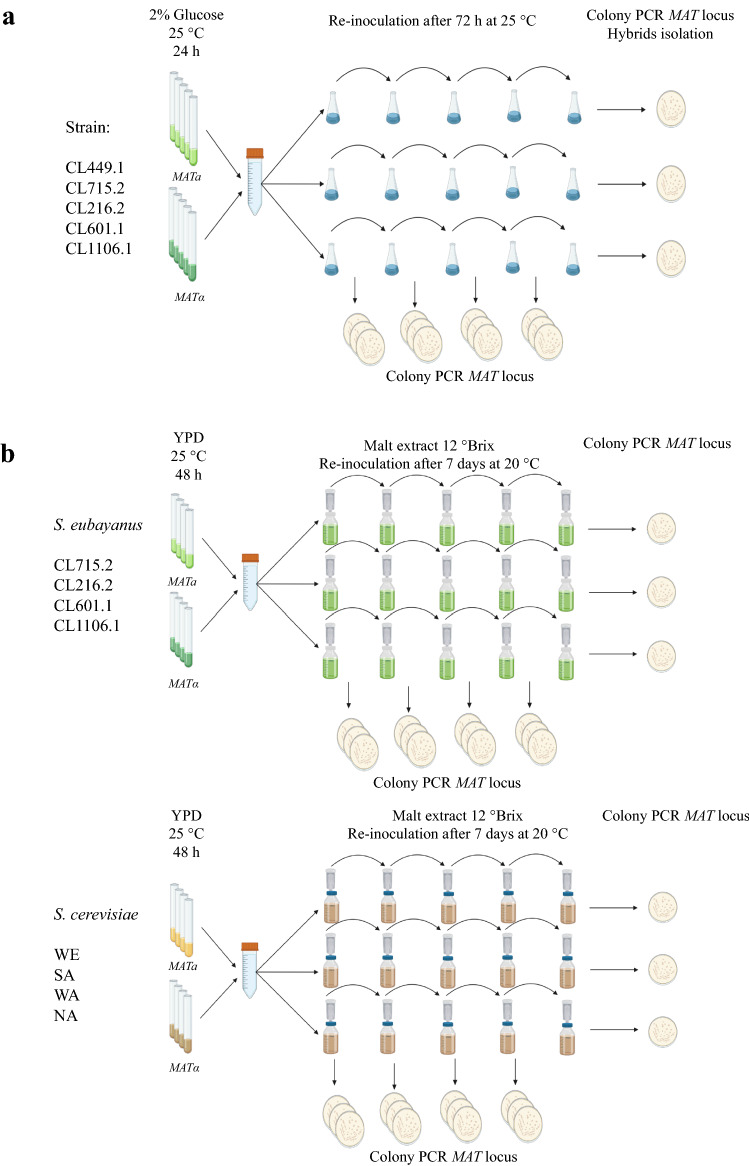
Experimental procedures to generate intraspecific hybrids. (**a**) Generation of intraspecific hybrids in synthetic medium. (**b**) Generation of intraspecific hybrids in malt extract. Created by BioRender.com.

**Figure 5:**
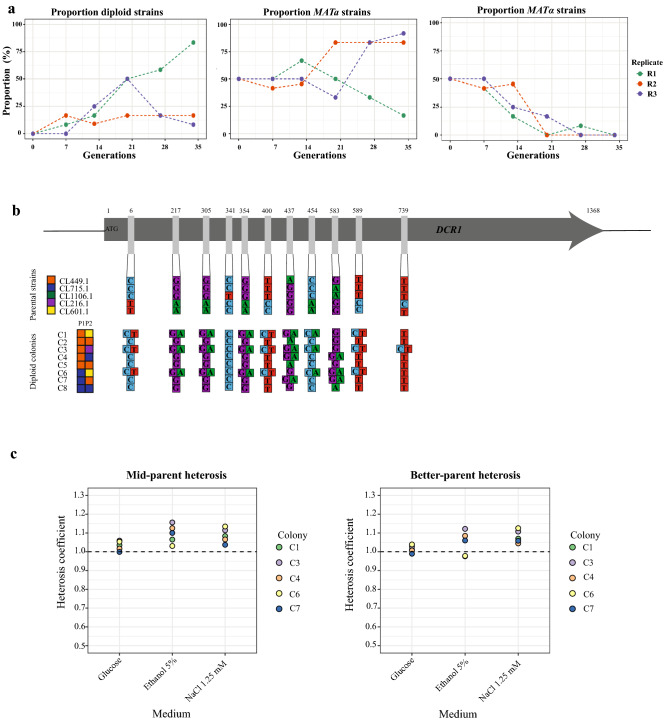
Generation of intraspecific hybrids in synthetic defined medium. (**a**) Proportion of diploid and haploid colonies identified during 34 generations. (**b**) SNPs identified in the eight diploid colonies genotyped for the *DCR1* gene; colored squares represent the different parental strains and the hybrid’s genotype. (**c**) Mid-parent (left) and best-parent heterosis (right) in intraspecific hybrids evaluated under three different environmental conditions (2% glucose, 5% ethanol and 1.25 mM NaCl).

Finally, we sought to evaluate the utility of our haploid collection by conducting, as proof of concept, two approaches to generate intra-specific hybrids (Fig. 4). First, we selected five strains that are representative of the different *S. eubayanus* lineages (CL449.1, CL715.1, CL216.1, CL601.1 and CL1106.1). The “*Mat*α” and “*Mat*a” versions of each strain were mixed in equal proportions and grown for 34 generations (four transfers) in a defined medium, and the proportion of haploid and diploid cells determined after each transfer (Fig. 4a). We evaluated the generation of diploid strains by colony isolation and identification of mating-type after each transfer. We observed a drastic decrease in *MATα* colonies in all three replicates. In replicates 2 and 3, we observed a predominance of *MATa* colonies, while a predominance of diploid colonies was observed in replicate 1 (Fig. 5a). Interestingly, *MATa* and *MATα* haploid strains did not exhibit growth rate differences under similar microculture conditions, and therefore the differences observed are specific under the dynamic competitive environment. A selective advantage for a certain trait is strongly dependent on environmental dynamics, where antagonism or competition can impact a strain’s success^70^. Previous studies demonstrated that sterile haploid mutants could exhibit a growth advantage over wild-type haploid cells^71^ suggesting that expression of mating pathway genes could differently impact the fitness of *MATa* and *MATα* cells.

The diploid colonies in replicate 1 could originate from crosses between different strains, or crosses between two opposite mating types of the same genetic background. In addition, diploid colonies obtained from the same replicate may be clones from the same single hybridization event. To pinpoint the source of this variation, we genotyped the polymorphic *DCR1* gene in eight diploid colonies to identify their respective parental strains (Fig. 5b and Supplementary Table S7). Five out of eight diploid colonies were identified as intraspecific hybrids: Three hybrids from replicate 1 (colony numbers 1, 3 and 4) and two from replicate 2 (colony numbers 6 and 7). Interestingly, in replicate 1 the CL449.1 strain predominated in both types of colonies, that is intraspecific hybrids and in self-crosses between strains of the same genetic background. On the other hand, CL715.1 strain predominated in replicates 2 and 3 in the two intraspecific hybrids (Supplementary Table S7).

To quantify the fitness of the hybrid-diploid colonies after the final serial transfer, we evaluated five, two and one diploid colonies from replicate 1, 2 and 3, respectively, under microculture conditions in 2% glucose, 5% ethanol and 1.25 mM NaCl, comparing their fitness against the five parental strains (Supplementary Table S8). We observed a significantly higher average growth rate (*p*-value < 0.05, ANOVA) in diploid colonies compared to parental strains in the three conditions evaluated, suggesting a greater fitness in these diploid individuals. Other diploid *Saccharomyces* intraspecific hybrids have shown superior phenotypes than their diploid parental strains, representing a successful strategy to survive under challenging conditions by creating phenotypic diversity^72–76^. Finally, we evaluated the heterosis coefficient in the five intraspecific hybrids relative to their respective parental strains in the same three conditions. In this case, we observed mild positive mid-parent and best-parent heterosis (heterosis coefficient > 1) (Fig. 5c). These results agree with previous studies, where intraspecific hybrids showed low levels of mid-parent heterosis for relative competitive growth, with no significant relationship between genetic divergence and heterosis^77^.

**Figure 6:**
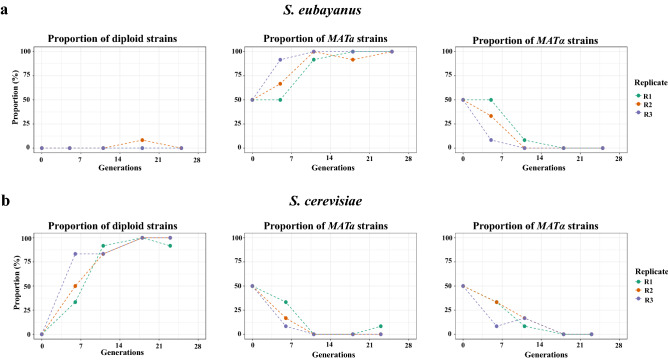
Generation of intraspecific hybrids under beer wort fermentation in S*. eubayanus* and *S. cerevisiae*. (**a**) Proportions of diploid and haploid colonies of *S. eubayanus* identified during 34 generations. (**b**) Proportions of diploid and haploid colonies of *S. cerevisiae* identified during 34 generations.

Considering the low intraspecific hybridization rate in *S. eubayanus*, we evaluated whether this situation depended on environmental conditions or the *Saccharomyces* species. For this, we carried out a second experimental approach for the generation of intraspecific hybrids by mass-mating under beer wort, as a representative alternative environmental condition. Furthermore, to determine whether the low hybridization rate was specific to *S. eubayanus* or reflected a more general trend in the *Saccharomyces* genus, we evaluated the hybridization rate in *S. cerevisiae* compared to *S. eubayanus* (Fig. 4b). We selected four strains representative of the PB-2, PB-3 and ADM lineages (CL715.1, CL216.1, CL601.1 and CL1106.1) in *S. eubayanus*, and four strains representative of the Wine/European, Sake, West African and North America lineages (DBVPG6765, Y12, DBVPG6044 and YPS128) in *S. cerevisiae*. The “*MATα*” and “*MATa*” versions of each strain were mixed and grown in malt extract for seven days and for 34 generations (four transfers). Subsequently, we determined the proportion of haploid and diploid cells after each transfer by colony PCR (Fig. 4b). As previously observed, in *S. eubayanus*, we identified a drastic decrease in *MATα* colonies, a predominance of *MATa* colonies, and the lack of hybrids after the fourth transfer in the three replicates (Fig. 6a)*.*

On the other hand, *S. cerevisiae* quickly returns to a diploid state, which represent more than 50% of the colonies analyzed after the second transfer (Fig. 6b). These results suggest that the intraspecific hybridization rate depends on environmental conditions and the *Saccharomyces* species. The predominance of haploid colonies of *S. eubayanus* may be due to several factors. The presence of colonies of both mating-types after the first transfer, without the detection of diploid colonies, suggests that indeed the different strains of *S. eubayanus* are not mating, unlike in *S. cerevisiae* where after the first transfer, at least 30% of the colonies were diploid (Fig. 6b). Previous studies have demonstrated that cells of different mating types grown in a liquid medium can optimize their decision to mate or to proliferate by detecting the ratio of opposite-sex partners to same-sex competitors^78^. Furthermore, specific sexual aggregation via α/a-agglutinins is required for mating in a suspension of cells in liquid, which is determined by the probability of random mating encounters and the interaction strength of sexual α/a-agglutinins^78,79^. Indeed, the production of pheromones and the sexual aggregation capacity of *S. eubayanus* have not been studied; both factors can vary between strains, may be different from those reported for other *Saccharomyces* species, and may impact their mating capacity^78,79^. Finally, another factor to consider is the adaptive advantage of the ploidy level to different environments, which has been a matter of interest in studies of ecology and evolution^80^. Previous research has shown some adaptive advantages of haploid cells compared to their isogenic diploid strain under nutritional stress conditions^61,81–84^.

## Conclusions

We obtained an extensive collection of haploid strains of *S. eubayanus* from different genetic backgrounds complementing previous resources generated for the species and the *Saccharomyces* genus. These strains were comprehensively phenotyped and represent a valuable and promising resource for studies in different aspects of biology, particularly for molecular genetic studies. Genomic analysis demonstrated the extremely low frequency of off-targets and no structural variants derived from the meiotic event. Finally, mass-mating experiments demonstrate the pertinence of the haploid collection for genetic studies and the preference of *S. eubayanus* to remain in the haploid state compared to *S. cerevisiae*, which quickly returns to a diploid state.

## Methods

### Strains

The *S. eubayanus* strains used in this study were selected from a collection of strains that have been published previously^36,37^. The strains comprised 83 Chilean strains collected from ten different localities^36^, one Argentinian strain^41^ and five North American strains^45^ (Supplementary Table S1). All strains were maintained on YPD solid medium (1% yeast extract, 2% peptone, 2% glucose, 2% agar). Yeast haploid strains are available upon request to the corresponding author.

### Generation of null mutants by CRISPR-Cas9

*HO* null mutants were generated using a CRISPR-Cas9 method^22^ to generate stable haploid strains. The guide RNAs (gRNAs) were designed using the *HO* coding sequences from the reference strain CBS12357^55^ and the Benchling online tool (https://www.benchling.com/). We selected the four gRNAs with the highest “on-target” score, an optimized score for 20 bp gRNAs with NGG PAM, developed by Doench et al*.*^64^. The gRNAs were synthesized as single-stranded oligonucleotides (Macrogen, Korea), adding the SapI overhangs, 5’-ATC and 5’-AAC, and were phosphorylated (10 µM final concentration) using 10 units of T4 polynucleotide kinase (New England BioLabs, NEB) in a final volume of 10 µL at 37 °C for 1 h. Double-stranded oligonucleotides were generated by annealing equimolar amounts of phosphorylated single-stranded oligonucleotides under the following conditions: denaturation at 96 °C for 6 min and then cooling to 23 °C with a ramp of 0.1 °C/s. The four gRNAs were separately cloned in the plasmid pAEF5^54^ (a gift from Gilles Fischer, Addgene plasmid #136,305), using standard “Golden Gate Assembly”^85^, digesting the plasmid with 10 units of SapI (NEB) and ligating this to each gRNA using 400 units of T4 DNA ligase (Promega) in a 10 µL final volume under the following conditions: 10 cycles at 42 °C for 2 min and 16 °C for 5 min, one cycle at 65 °C for 10 min and one cycle at 80 °C for 10 min. The product obtained was transformed into the *E. coli* DH5alpha competent (NEB, UK) following the manufacturer’s recommendations. The plasmids were recovered using a FavorPrep Plasmid DNA Extraction Mini Kit (Favorgen Biotech Corp.), quantified using a Qubit system (Thermo Fisher, Waltham, Massachusetts, US.) and verified in a 1.5% agarose gel.

The donor DNA consisted of a 100 bp synthetic double-stranded DNA fragment (Macrogen, Korea) containing flanking sequences to the target *HO* gene, with a sequence homology of 50 bp upstream of the start codon and 50 bp downstream of the stop codon. This donor DNA was amplified by PCR using 1 × Phusion High-Fidelity PCR Master Mix (Thermo Fisher), 0.52 µM of each primer and 4 µM of donor DNA under the following conditions: 98 °C for 30 s; 35 cycles at 98 °C for 10 s, 55 °C for 30 s and 72 °C for 20 s; 72 °C for 15 min.

Finally, each diploid strain was co-transformed with the plasmid carrying the gRNA together with the Cas9 gene, and the donor DNA using the standard lithium acetate protocol^86^ with temperature modifications. Briefly, the strains were grown overnight in 5 mL YPD under constant agitation at 20 °C. Then, 300 µL overnight culture were inoculated in 5 mL YPD and incubated under the same conditions for 4 h or until the culture reached 10^8^ cells/mL. From this, 1 mL was washed three times with distilled water, then washed twice in 1 mL of lithium acetate 0.1 M (LiAc) and cells were recovered by centrifugation. The pellet of cells was mixed with 240 µL polyethylene glycol 3350 50% w/v (PEG 3350), 36 µL 1 M LiAc, 20 µL denatured salmon sperm DNA (10 mg/mL), 500 ng of a plasmid carrying the gRNA and the Cas9, and 24 µL of donor DNA for the repair of CRISPR-induced DSBs. The cells were resuspended by vortex mixing and incubated for 45 min at 20 °C. Then, a heat shock was performed at 37 °C for 20 min. After transformation, cells were washed with 1 mL distilled water and plated on YPD with 0.2 mg/mL hygromycin for selection. Plates were incubated between 3 to 5 days at 20 °C. Correct gene deletion was confirmed by colony PCR using GoTaq DNA polymerase (Promega). All primer, gRNAs and donor DNA sequences are listed in Supplementary Table S9.

### Generation of stable haploid strains

Isogenic haploid strains were constructed by deleting the *HO* gene using a CRISPR-Cas9 method as described above. Initially, the four gRNAs were evaluated separately in the CL216.1 strain, determining the efficiency of each gRNA as the number of colonies with the proper deletion divided by the total number of colonies evaluated by colony PCR. The most efficient gRNA was used to transform the other strains in the collection. Diploid *Δho* cells were sporulated on 2% potassium acetate agar plates (2% agar) for at least seven days at 20 °C. Meiotic segregants were obtained by dissecting tetrad ascospores treated with 10 µL Zymolyase 100 T (50 mg/mL) on YPD agar plates with a SporePlay micromanipulator (Singer Instruments, UK). Spores from four viable spore tetrads were selected to determine the mating type by colony PCR of the *MAT locus*^8^ using GoTaq DNA Polymerase (Promega). All the primers used in these experimental procedures are listed in Supplementary Table S9.

### Spore viability

Strains were sporulated on 2% potassium acetate agar plates as described above. Tetrad ascospores were treated with 10 µL Zymolyase 100 T (50 mg/mL) and dissected with a SporePlay micromanipulator. At least 20 ascospores per strain were dissected on YPD plates and incubated at 20 °C for four days. Spore viability was calculated using the formula (n° of viable spores/n° of dissected spores) × 100.

### Genotypic characterization

Genomic DNA from a single spore of CL216.1-a, CL715.1-a and CL601.1-a was prepared for whole-genome sequencing using the Qiagen Genomic-tip 20/G kit (Qiagen, Germany) as previously described^36^ and sent for DNBseq sequencing (BGISEQ-G400 platform). Reads were filtered and trimmed using Fastp 0.20.1 (–3-l 50-cut_mean_quality 30)^87^. Cleaned reads were mapped to the *S. eubayanus* reference strain CBS12357 using BWA mem 0.7.17^88^, after which PCR duplicates were marked using Picard tools. Mapping files corresponding to the original diploid strains were obtained from^36^. Variants were jointly called on haploid and diploid alignments using freeBayes 1.3.1^89^. Variants were analyzed in R and visually inspected using the IGV genome browser^90^.

To perform nanopore sequencing of a full tetrad of the CL216.1 strain, DNA was extracted from each spore using the Quick-DNA HMW MagBead Kit (Zymo Research). High molecular weight genomic DNA was purified using magnetic beads following the kit manufacturer’s protocol. DNA integrity was verified using an agarose gel and DNA quantity was assessed using Quantus (Promega, Madison, Wisconsin, US.). Libraries were prepared following the manufacturer's protocol, barcodes were added to each library, and these were sequenced on a single R9.4 flow cell using a Minion (Oxford Nanopore Technologies, UK). The raw fast5 files were transformed to fastq files and de-barcoded using Guppy v5.0.14 with the “super high accuracy” model. Long reads were mapped to the *S. eubayanus* reference CBS12357 with minimap2 (ax map-ont –secondary = no)^91^ and variants were called using PEPPER (–ont_r9_guppy5_sup)^92^. Long-read assemblies of each spore were generated using Flye v2.9 (–nano-hq –scaffold)^93^. To evaluate synteny, each assembly was compared using nucmer^94^. Structural variations were identified using MUM&Co^95^.

### Phenotypic characterization

One *MATa* and *MATα* haploid version of each haploid strain and their respective diploid parental background were phenotyped under microculture conditions. For this, we estimated mitotic growth in 96-well plates under different conditions in three biological replicates, including: Yeast Nitrogen Base (YNB) supplemented with 2% glucose at 25 °C, 30 °C and 34 °C; 2% glycerol, 2% glucose and 1.25 mM NaCl; 2% maltose; 2% glucose and 5% ethanol; 2% glucose and 10% ethanol. All conditions were evaluated at 25 °C in 0.67% YNB medium (Difco, France). First, haploid and diploid strains were pre-grown in 200 µL YNB medium supplemented with 2% glucose for 48 h at 25 °C and used to inoculate a 96-well plate with a final volume of 200 µL at an initial OD_600nm_ of 0.1. The growth curves were monitored by measuring the OD_600nm_ every 30 min in a Tecan Sunrise absorbance microplate reader (Tecan Group Ltd., Switzerland). All experiments were carried out in triplicates. The maximum specific growth rate (µ_max_) was determined using GrowthRates software with default parameters^96^.

Fermentations were carried out in three biological replicates using previously oxygenated (15 mg/L) 12°Plato (°P) beer wort, supplemented with 0.3 ppm ZnCl_2_. The pre-cultures were grown in 5 mL 6°P wort for 24 h at 20 °C in constant agitation at 150 rpm. Inocula were then transferred to 50 mL 12°P wort and incubated for 24 h at 20 °C in constant agitation at 150 rpm. The cells were collected by centrifugation and used to calculate the final cell concentration for use in each fermentation according to the formula described by White and Zainasheff^97^. Cells were inoculated into 50 mL 12°P wort in 250 mL bottles and airlocks with 30% glycerol. The fermentations were incubated at 12 °C, with no agitation for 15 days and monitored by weighing the bottles daily to determine weight loss over time.

### Metabolites quantification by HPLC

Glucose, fructose, maltose, maltotriose and ethanol concentrations were determined by High-Performance Liquid Chromatography (HPLC) after 15 days of fermentation as previously described^35,48^. Samples were obtained extracting 0.5 mL fermented beer wort and filtered using 0.22 μm filters. Filtered samples (20 µL) were injected in a Shimadzu Prominence HPLC (Shimadzu, USA) with a BioRad HPX-87H column using 5 mM sulfuric acid and 4 mL acetonitrile per liter of sulfuric acid as the mobile phase at a 0.5 mL/min flow rate^98^. Glucose, fructose, maltose and maltotriose uptake were estimated as the difference between the initial and final concentration before and after fermentation, respectively.

### Generation of intra-specific hybrids by mass-mating

Five different genetic backgrounds were selected to generate intra-specific hybrids by mass-mating (CL216.1, CL601.1, CL715.1, CL1106.1 and CL449.1) (Fig. 4a). First, one colony from each *S. eubayanus* strain and mating type was cultured in 0.67% YNB medium (Difco, France) with 2% glucose at 25 °C in constant agitation at 150 rpm. Each pre-inoculum was then utilized to prepare a co-culture at an initial OD_600nm_ of 0.1 of each strain in 150 mL 0.67% YNB medium with 2% glucose. This co-culture was divided in three 50 mL replicates and incubated at 25 °C in constant agitation at 150 rpm during 72 h. Subsequently, the cultures were used to inoculate fresh 50 mL cultures at an initial OD_600nm_ of 0.1, and this procedure was sequentially repeated four times. After each incubation, colonies were isolated for each replicate on YPD solid medium and the mating type determined by colony PCR as described above (at least 10 colonies per replicate). The number of generations was determined using the formula log_2_(OD_600 final_/0.1)^99^.

Diploid colonies identified after the last incubation were characterized under microculture conditions as described above, together with the parental diploid and haploid strains. The conditions evaluated were: 2% glucose at 25 °C, 2% glucose with 1.25 mM NaCl, and 2% glucose with 5% ethanol. Mid-parent and best-parent heterosis were determined as previously described^77,100^, using Eqs. (1) and (2), where mid-parent heterosis denotes the hybrid deviation from the mid-parent performance and best-parent heterosis denotes the hybrid deviation from the better parent phenotypic value^101^.

1 $$Mid-parent\, heterosis= \frac{{\mu max}_{h}}{\overline{{\mu max }_{p}}}$$

2 $$Best-parent \,heterosis= \frac{{\mu max}_{h}}{{\mu max}_{bp}}$$

where:

${\mu max}_{h}$: growth rate of a hybrid.

$$\overline{{\mu max }_{p} }= \frac{{\mu max}_{parental 1}+ {\mu max}_{parental 2}}{2}$$

$${\mu max}_{bp}=\mathrm{max}({\mu max}_{parental 1}, {\mu max}_{parental 2})$$

Intra-specific hybrids by mass-mating were also generated under fermentation conditions (Fig. 4b). Four different genetic background were selected: CL216.1, CL601.1, CL715.1 and CL1106.1. The pre-culture conditions were the same as described above; each haploid strain was grown in 5 mL 6°P wort for 24 h at 20 °C in constant agitation at 150 rpm, then transferred to 50 mL 12°P wort and incubated for 24 h at 20 °C in constant agitation at 150 rpm. Each pre-inoculum was used to prepare a co-culture at a final concentration of 1 × 10^6^ cells/mL and transferred to three replicates to obtain a final concentration of 1.5 × 10^6^ cells/mL in 50 mL 12°P wort in 250 mL bottles and airlocks with 30% glycerol. The fermentations were incubated at 20 °C, with no agitation for 7 days and monitored by weighing the bottles daily to determine weight loss over time. At the end of fermentation, the cultures were used to inoculate fresh 50 mL 12°P wort at an inoculum density of 1.5 × 10^6^ cells/mL, repeating the fermentation and re-inoculation four times. Also, we used four different *S. cerevisiae* strains that represent the main lineages described in the species: YPS128 (North American, NA), DBVPG6044 (West African, WA), Y12 (Sake, SA) and DBVPG6765 (Wine/European, WE) (Supplementary Table S10)^8^. The number of generations was determined using the formula log_2_(final cells/1.5 × 10^6^)^102^. Finally, after each fermentation, the proportions of diploid and haploid cells were determined by colony PCR of colonies isolated for each replicate on solid YPD medium, as described above.

### Hybrid genotyping

Diploid colonies identified after the last incubation in the mass-mating experiment were genotyped to determined their intraspecific hybrid status by sequencing the *DCR1* gene^45^. Genomic DNA of eight diploid colonies was extracted using the Wizard® Genomic DNA Purification Kit (Promega) according to the manufacturer’s instructions. PCR reactions were performed in a final volume of 25 µL containing 1 × GoTaq DNA polymerase Master Mix (Promega), 0.2 µM of each primer and 1 µL DNA (25 ng/µL) under the following conditions: 95 °C for 2 min; 35 cycles at 95 °C for 15 s, 52–55 °C for 15 s and 72 °C for 90 s; 72 °C for 5 min. Sanger sequencing was performed in an Applied Biosystem 3130XL instrument (Pontificia Universidad Católica de Chile, Santiago, Chile). Sequences of diploid colonies and wild strains were aligned using a Multiple Sequence Comparison by Log-Expectation (MUSCLE) algorithm with default parameters in MEGA-X version 10.2.4. Sequences of wild strains used in the mass-mating experiment were previously published^36^.

### Data and statistical analysis

Data visualization and statistical analyses were performed with R software version 4.0.3. The maximum specific growth rates and total CO_2_ loss were compared using an analysis of variance (ANOVA) and the mean values of the three replicates were statistically analyzed using Student’s t-test and corrected for multiple comparisons using the Benjamini–Hochberg method. A *p-value* less than 0.05 (*p* < 0.05) was considered statistically significant. A principal component analysis (PCA) was performed using the FactoMineR package version 2.4 to compute principal component methods and the factoextra package version 1.07 for extracting, visualizing and interpreting the results. Heatmaps was generated using the ComplexHeatmap package version 2.6.2.

## Supplementary Information

Supplementary Information 1.

Supplementary Information 2.

Supplementary Information 3.

Supplementary Information 4.

Supplementary Information 5.

Supplementary Information 6.

Supplementary Information 7.

Supplementary Information 8.

Supplementary Information 9.

Supplementary Information 10.

Supplementary Information 11.

Supplementary Information 12.

Supplementary Information 13.

## Data Availability

All fastq sequences were deposited in the National Center for Biotechnology Information (NCBI) as a Sequence Read Archive under the BioProject accession number PRJNA800259 (www.ncbi.nlm.nih.gov/bioproject/800259).
