# Supplementary material for: A Saccharomyces eubayanus haploid resource for research studies
Source: Sci Rep. 2022 Apr 8;12:5976. doi: 10.1038/s41598-022-10048-8 (PMC8993842; doi:10.1038/s41598-022-10048-8)
Supplement: Supplementary file 1 — Supplementary Information 1. [file 41598_2022_10048_MOESM1_ESM.pdf]

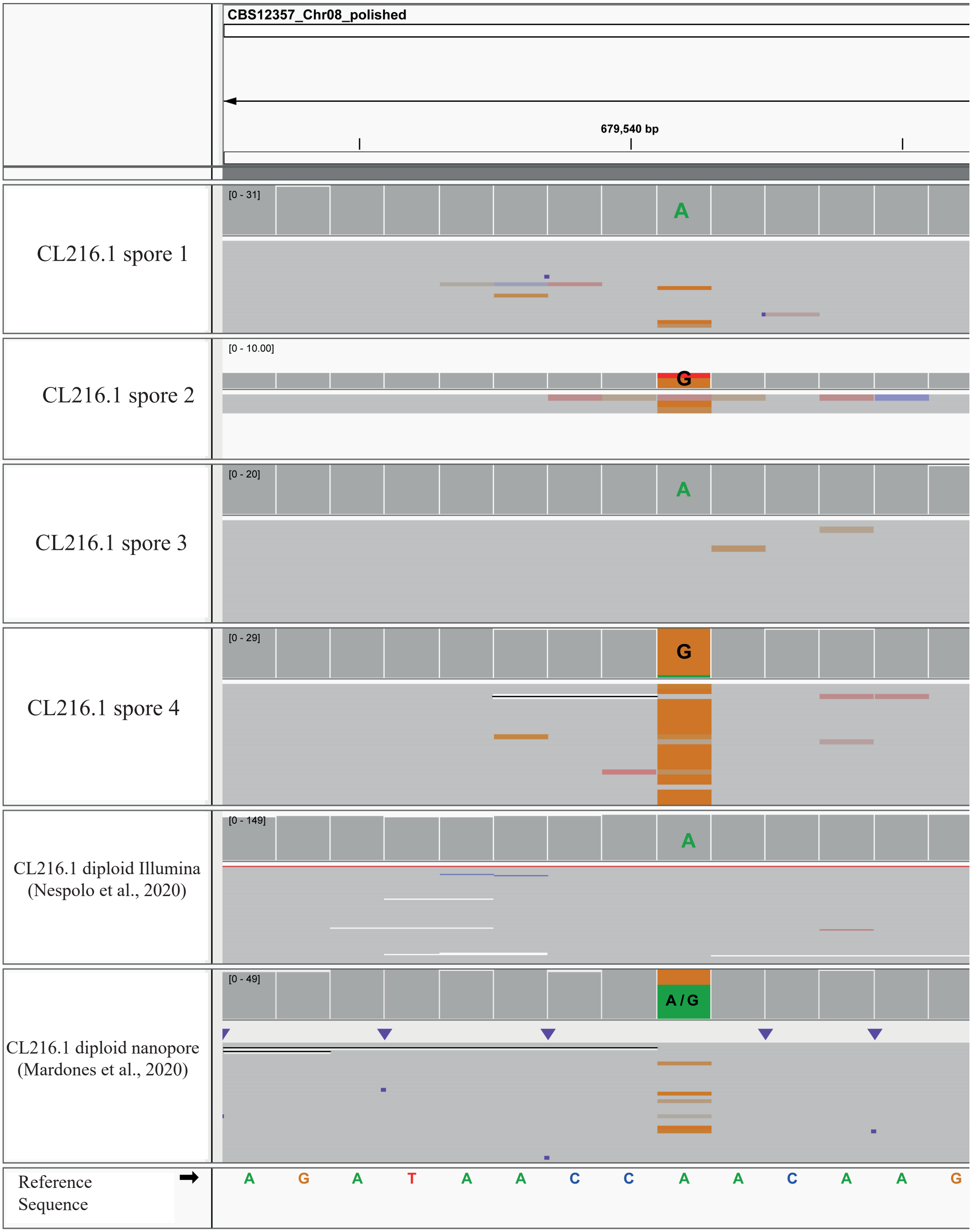

Supplementary Figure S1. Read alignments to the *S. eubayanus* reference genome.
Samples correspond to four haploid spores of CL216.1, a diploid CL216.1 sequenced with short reads (Illumina sequencing), and a diploid CL216.1 sequenced with long reads (Nanopore sequencing).
A potentially novel heterozygous site (A/G) is shown in diploid CL216.1 strain (Nanopore) which segregates in a 2:2 proportion among the four CL216.1 spores.
