# Supplementary material for: A Saccharomyces eubayanus haploid resource for research studies
Source: Sci Rep. 2022 Apr 8;12:5976. doi: 10.1038/s41598-022-10048-8 (PMC8993842; doi:10.1038/s41598-022-10048-8)
Supplement: Supplementary file 2 — Supplementary Information 2. [file 41598_2022_10048_MOESM2_ESM.pdf]

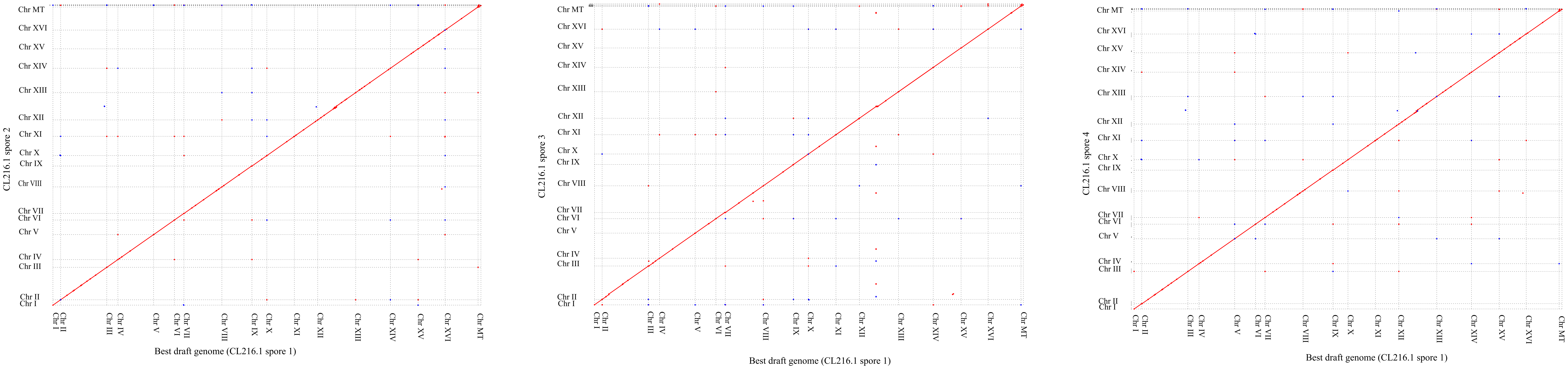

Supplementary Figure S2. Dot plot representation of DNA sequence identity between CL216.1 spore 1 with spore 2, spore 3 and spore 4.
