# Supplementary material for: A Saccharomyces eubayanus haploid resource for research studies
Source: Sci Rep. 2022 Apr 8;12:5976. doi: 10.1038/s41598-022-10048-8 (PMC8993842; doi:10.1038/s41598-022-10048-8)
Supplement: Supplementary file 3 — Supplementary Information 3. [file 41598_2022_10048_MOESM3_ESM.docx]

**Supplementary information**

**Supplementary Figure legends**

**Supplementary Figure S1.** Read alignments to the *S. eubayanus* reference genome. Samples correspond to four haploid spores of CL216.1, a diploid CL216.1 sequenced with short reads (Illumina sequencing), and a diploid CL216.1 sequenced with long reads (Nanopore sequencing). A potentially novel heterozygous site (A/G) is shown in diploid CL216.1 strain (Nanopore) which segregates in a 2:2 proportion between the four CL216.1 spores.

**Supplementary Figure S2**. Dot plot representation of DNA sequence identity between CL216.1 spore 1 with spore 2, spore 3 and spore 4.

**Supplementary Table legends**

**Supplementary Table S1.** *S. eubayanus* strains used in this study.

**Supplementary Table S2.** Phenotype data (μ_max_) for parental and haploid strains.

**Supplementary Table S3.** Sugar consumption and ethanol production during beer wort fermentation.

**Supplementary Table S4.** Illumina mapping statistics. M refers to Millions.

**Supplementary Table S5.** (**a**) Putative off-target mutations generated during HO deletion by CRISPR-Cas9 on CL601.1, CL216.1 and CL715.1 strains. (**b**) Heterozygous calls in diploid and haploid strains of CL715.1. (**c**) Heterozygous calls in diploid and haploid strains of CL216.1. (**d**) Heterozygous calls in diploid and haploid strains of CL601.1.

**Supplementary Table S6.** Nanopore Assembly statistics.

**Supplementary Table S7.** SNPs identified in the eight diploid colonies by *DCR1* genotyping.

**Supplementary Table S8.** Phenotype data (μ_max_) for parent and hybrid strains.

**Supplementary Table S9.** Primers used in this study.

**Supplementary Table S10.** *S. cerevisiae* strains used in this study.
